# Lepromatous Leprosy Mimicking Sarcoidosis With Elevated Serum ACE Levels: A Deceptive Granulomatous Presentation

**DOI:** 10.7759/cureus.108374

**Published:** 2026-05-06

**Authors:** Gokul Prashanth, Dhanasekar T

**Affiliations:** 1 Pulmonary Medicine, Sri Ramachandra Institute of Higher Education and Research, Chennai, IND

**Keywords:** ace serum levels, chronic granulomatous disease, cutaneous sarcoidosis, lepromatous leprosy, skin nodules, tuberculosis masquerading

## Abstract

Granulomatous diseases often present with overlapping clinical and histopathological features, posing a diagnostic challenge, particularly in regions endemic for tuberculosis. We report the case of a 68-year-old diabetic male with a significant smoking history who presented with progressive shortness of breath and cough for almost three weeks' duration and multiple cutaneous nodules, present for six years, that increased in size and number over two months before presentation. Initial evaluation revealed normal chest imaging, negative microbiological tests for tuberculosis, a non-reactive Mantoux test, and elevated serum angiotensin-converting enzyme (ACE) levels, raising suspicion for the presence of granulomatous disease, like cutaneous tuberculosis, sarcoidosis, and other non-tuberculosis mycobacteria.

However, further evaluation, including dermatological assessment and histopathological examination of skin lesions, which demonstrated foamy histiocytic infiltration, was performed. Wade-Fite staining revealed numerous acid-fast bacilli with a high bacterial index, confirming the diagnosis of lepromatous leprosy. The patient was initiated on multidrug therapy with rifampicin, dapsone, and clofazimine, with close follow-up. This case highlights the importance of maintaining a broad differential diagnosis in granulomatous diseases and emphasizes the need for tissue diagnosis before initiating empirical therapy, especially in tuberculosis-endemic settings.

## Introduction

Granulomatous inflammation is a form of chronic inflammatory response characterized by the aggregation of activated macrophages, forming epithelioid histiocytes and multinucleated giant cells. In countries with a high burden of tuberculosis, granulomatous disease is frequently attributed to *Mycobacterium tuberculosis*, and patients are often started on empirical antitubercular therapy based on clinical and radiological suspicion [1,2].

This pattern may be seen in a wide spectrum of conditions, including infectious diseases such as tuberculosis and leprosy, as well as non-infectious disorders like sarcoidosis [3,4]. However, several other conditions can mimic tuberculosis, both clinically and histologically, leading to potential misdiagnosis and inappropriate treatment, for example, *Nocardia* species, *Pneumocystis jiroveci*, *Echinococcus granulosus*, *Toxoplasma gondii*, *Actinomyces* species, and others [5]. Sarcoidosis is another important cause of granulomatous inflammation, typically characterized by non-caseating granulomas and supported by clinical, radiological, and laboratory findings such as elevated serum angiotensin-converting enzyme (ACE) levels [3]. However, these features are not entirely specific and may overlap with other granulomatous disorders.

Leprosy, caused by *Mycobacterium leprae*, remains an important but often overlooked differential diagnosis, particularly in endemic regions [4,6]. Its cutaneous manifestations can mimic other granulomatous conditions, making clinicopathological correlation essential for accurate diagnosis. This report describes a case of lepromatous leprosy initially suspected to be sarcoidosis, highlighting the diagnostic challenges associated with granulomatous diseases.

## Case presentation

A 68-year-old diabetic male with a history of active smoking (more than 30 pack-years) presented with complaints of cough associated with scanty, mucoid expectoration and progressive shortness of breath over three to four weeks (modified Medical Research Council (mMRC) grade II-III). He also reported nasal obstruction and a sensation of nasal blockage present for almost a year that had increased in the last three months before presentation.

**Figure 1:**
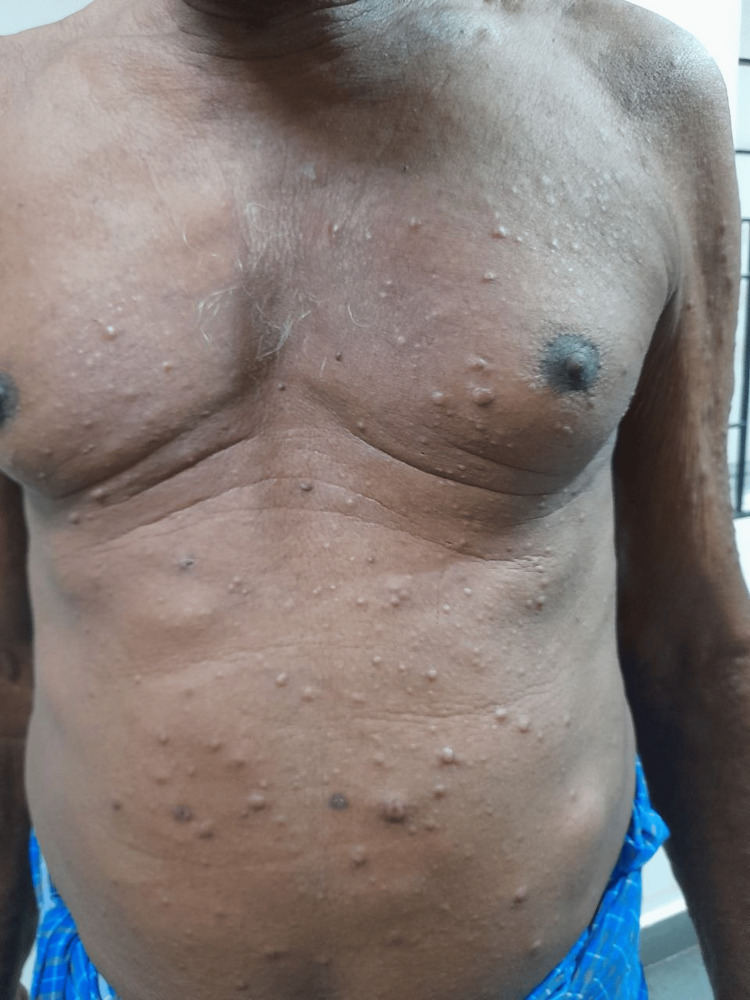
Skin nodules randomly distributed on the thorax and abdomen

**Figure 2:**
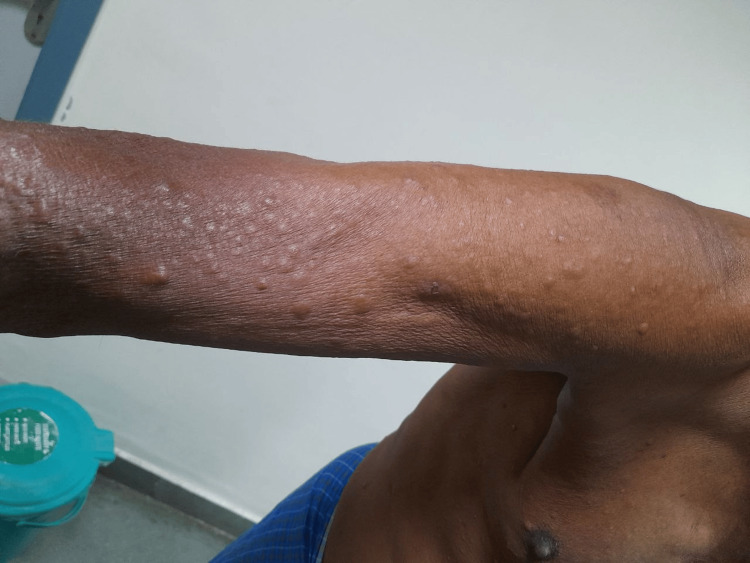
Skin nodules over the right arm and forearm

On examination, multiple hyperpigmented and erythematous infiltrated papules over the trunk were noted, and multiple non-tender cutaneous nodules were noted over the thorax, abdomen (Figure 1), and bilateral upper and lower limbs (Figure 2). These nodules were hard, with no sinus discharge and reduced sensation over the bilateral feet. Respiratory system examination was unremarkable. 

Chest radiography revealed normal lung fields. High-resolution CT of the thorax showed no significant parenchymal abnormalities, mediastinal lymphadenopathy, or pleural involvement. Spirometry demonstrated lung function within normal limits with adequate lung volume (Table 1).

**Table 1:** Pulmonary function test showing normal spirometry with no significant bronchodilator response The MEF25 is measured when only 25% of the FVC remains in the lungs, and MEF75 is measured when 75% of the FVC remains in the lungs. FEV1: Forced expiratory volume at the end of the first second, FVC: Forced vital capacity, FEV1/FVC: Ratio of the volume of the air expired in the first second to the total forced vital capacity, MEF: Maximum expiratory flow

Parameters	Lower limit normal	Pre-bronchodilator capacity	Reference %	Post-bronchodilator capacity	Reference %
Forced expiratory volume (FEV) 1	1.82	2.54	99	2.63	103
Forced vital capacity (FVC)	2.43	3.08	93	3.15	95
FEV1/FVC	55	82		83	
Maximum expiratory flow (MEF)25	0.09	0.86	71	0.96	80
MEF75	3.6	6.42	106	7.69	127

**Figure 3:**
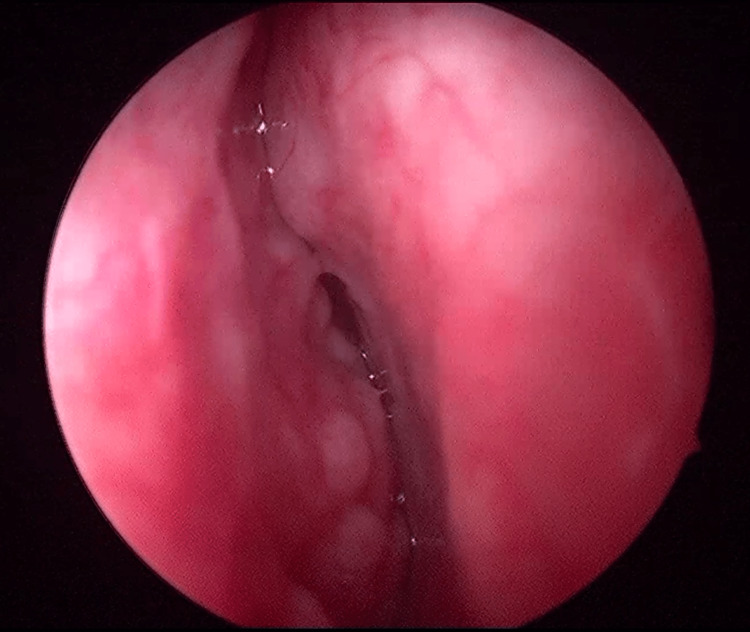
Diagnostic nasal endoscopy showing cobblestone pattern of nodules in bilateral mucosa

Microbiological evaluation, including sputum smear for acid-fast bacilli and nucleic acid amplification testing (GeneXpert MTB, Cepheid, Sunnyvale, CA, USA), was negative. The Mantoux test showed no induration. In view of the presence of cutaneous lesions, negative tubercular etiology, and negative Mantoux skin test, a differential diagnosis of cutaneous sarcoidosis was considered, and serum ACE levels were found to be elevated, while serum calcium and 24-hour urinary calcium levels were within normal limits. Ophthalmological evaluation did not reveal evidence of uveitis or choroidal tubercles. Given the nasal symptoms, an otorhinolaryngology consultation was obtained. Diagnostic nasal endoscopy revealed nodular mucosal lesions with a cobblestone appearance (Figure 3).

**Figure 4:**
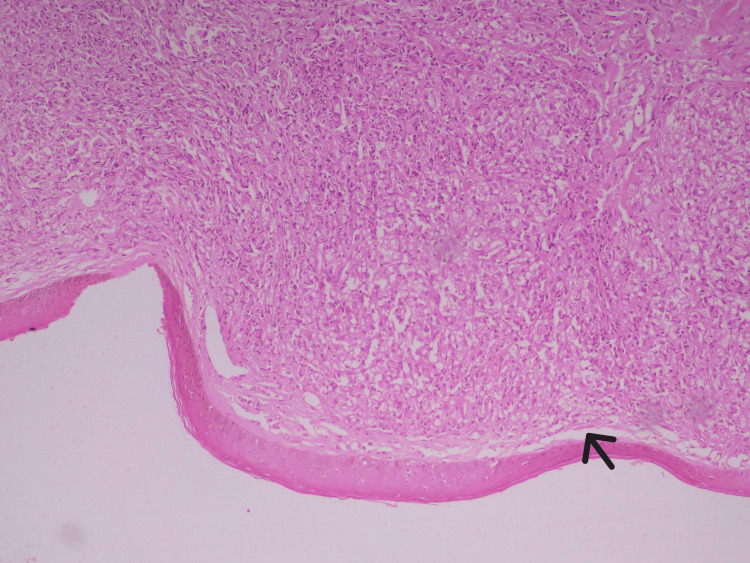
Histopathology of skin biopsy (low power field with magnification of 200x) Seen are thinned-out epidermis and epidermal-dermal junction in the Grenz zone or Band of Unna, and a narrow, clear band of collagen connective tissue located in the papillary dermis, separating the epidermis from an underlying dense and diffuse infiltrate of leprosy cells (black arrow).

**Figure 5:**
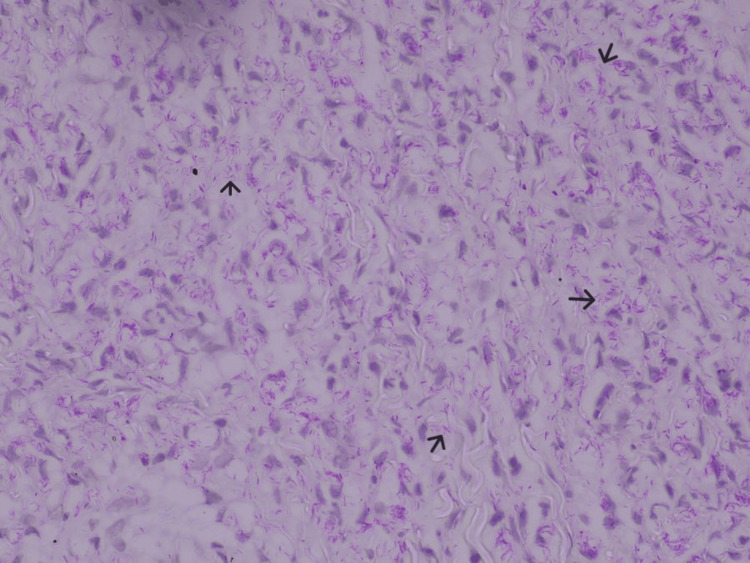
Histopathology of skin biopsy Wade Fite stain showing lepromatous bacilli in high power field at 400x magnification (black arrows)

A skin biopsy from the nodular lesions demonstrated a thinned epidermis with dermal infiltration by foamy histiocytes and epithelioid cells forming granulomatous aggregates (Figure 4). Wade-Fite staining was strongly positive for acid-fast bacilli, with a bacterial index of 5 (100-1000 bacilli per high-power field) (Figure 5).

Based on these findings, a diagnosis of lepromatous leprosy was established. Peripheral nerve examination revealed non-thickened and non-tender ulnar and radial cutaneous nerves, and greater auricular nerves were visible on both the right and left sides. The patient was initiated on multidrug therapy consisting of rifampicin, dapsone, and clofazimine and improved symptomatically. 

## Discussion

Leprosy is a chronic infectious disease caused by Mycobacterium leprae, primarily affecting the skin, peripheral nerves, and mucosal surfaces of the upper respiratory tract [4]. The clinical spectrum ranges from paucibacillary to multibacillary forms depending on the host's immune response.

The diagnostic challenge in this case stemmed from overlapping features with more commonly encountered granulomatous diseases. The initial differential diagnoses included tuberculosis and cutaneous sarcoidosis in view of elevated ACE levels and a negative Mantoux skin test. Tuberculosis was considered, given its high prevalence in endemic settings and the patient’s respiratory symptoms. However, the absence of radiological abnormalities, negative sputum studies including acid-fast bacilli smear and nucleic acid amplification testing, and a non-reactive Mantoux test made this diagnosis less likely [1,2].

Lepromatous leprosy is an important mimicker that can resemble other granulomatous conditions, such as sarcoidosis, both clinically and through nonspecific laboratory findings [7,8]. Sarcoidosis was strongly suspected due to elevated serum ACE levels in the setting of a negative tuberculosis workup. However, the lack of characteristic radiological findings such as bilateral hilar lymphadenopathy, along with the absence of supportive systemic features including uveitis and hypercalcemia, argued against this diagnosis [3,9].

A key diagnostic clue in this patient was the presence of multiple cutaneous nodules, which were present for a long time, and the patient did not seek any medical management for the same until the patient developed nasal stiffness and shortness of breath due to the presence of nodules along the nasal mucosa. These findings prompted tissue evaluation, which proved decisive. Histopathological examination demonstrated diffuse dermal infiltration by foamy histiocytes with granulomatous aggregates, and Wade-Fite staining revealed numerous acid-fast bacilli with a high bacterial index, confirming the diagnosis of lepromatous leprosy.

This case underscores the importance of maintaining a broad differential diagnosis when evaluating granulomatous diseases, particularly in tuberculosis-endemic regions where there is a tendency to initiate empirical antitubercular therapy [1,2]. Failure to recognize this entity may lead to misdiagnosis and inappropriate treatment, including unnecessary exposure to antitubercular therapy or immunosuppressive agents. Despite advances in management, leprosy continues to pose diagnostic challenges in endemic regions such as India [6].

## Conclusions

Granulomatous diseases present significant diagnostic challenges due to overlapping clinical and laboratory features. In tuberculosis-endemic regions, there is a tendency to initiate empirical antitubercular therapy, which may lead to misdiagnosis and unnecessary drug exposure. This case highlights the importance of maintaining a broad differential diagnosis, including less commonly considered conditions such as leprosy. Whenever feasible, a definitive diagnosis should be established through tissue sampling with microbiological confirmation. Early tissue evaluation in atypical granulomatous presentations reduces diagnostic delay and avoids inappropriate empirical therapy. A multidisciplinary approach involving clinicians, pathologists, and microbiologists is essential in accurately diagnosing atypical presentations of granulomatous disease and ensuring appropriate management.
